# Diversity of *Melissococcus plutonius* from Honeybee Larvae in Japan and Experimental Reproduction of European Foulbrood with Cultured Atypical Isolates

**DOI:** 10.1371/journal.pone.0033708

**Published:** 2012-03-19

**Authors:** Rie Arai, Kiyoshi Tominaga, Meihua Wu, Masatoshi Okura, Kazutomo Ito, Naomi Okamura, Hidetaka Onishi, Makoto Osaki, Yuya Sugimura, Mikio Yoshiyama, Daisuke Takamatsu

**Affiliations:** 1 Saitama Prefectural Chuo Livestock Hygiene Service Center, Saitama, Japan; 2 The United Graduate School of Veterinary Sciences, Gifu University, Gifu, Japan; 3 Yamaguchi Prefectural Institute of Public Health and Environment, Yamaguchi, Japan; 4 Graduate School of Life and Environmental Sciences, University of Tsukuba, Tsukuba, Japan; 5 Bacterial and Parasitic Disease Research Division, National Institute of Animal Health, National Agriculture and Food Research Organization, Tsukuba, Japan; 6 Gifu Prefectural Gifu Livestock Hygiene Service Center, Gifu, Japan; 7 Animal Quarantine Service, Ministry of Agriculture, Forestry and Fisheries, Yokohama, Japan; 8 Fukushima Prefectural Kenchu Livestock Hygiene Service Center, Koriyama, Japan; 9 Honey Bee Research Unit, Animal Breeding and Reproduction Research Division, National Institute of Livestock and Grassland Science, National Agriculture and Food Research Organization, Tsukuba, Japan; East Carolina University School of Medicine, United States of America

## Abstract

European foulbrood (EFB) is an important infectious disease of honeybee larvae, but its pathogenic mechanisms are still poorly understood. The causative agent, *Melissococcus plutonius*, is a fastidious organism, and microaerophilic to anaerobic conditions and the addition of potassium phosphate to culture media are required for growth. Although *M. plutonius* is believed to be remarkably homologous, in addition to *M. plutonius* isolates with typical cultural characteristics, *M. plutonius*-like organisms, with characteristics seemingly different from those of typical *M. plutonius*, have often been isolated from diseased larvae with clinical signs of EFB in Japan. Cultural and biochemical characterization of 14 *M. plutonius* and 19 *M. plutonius*-like strain/isolates revealed that, unlike typical *M. plutonius* strain/isolates, *M. plutonius*-like isolates were not fastidious, and the addition of potassium phosphate was not required for normal growth. Moreover, only *M. plutonius*-like isolates, but not typical *M. plutonius* strain/isolates, grew anaerobically on sodium phosphate-supplemented medium and aerobically on some potassium salt-supplemented media, were positive for β-glucosidase activity, hydrolyzed esculin, and produced acid from L-arabinose, D-cellobiose, and salicin. Despite the phenotypic differences, 16S rRNA gene sequence analysis and DNA-DNA hybridization demonstrated that *M. plutonius*-like organisms were taxonomically identical to *M. plutonius*. However, by pulsed-field gel electrophoresis analysis, these typical and atypical (*M. plutonius*-like) isolates were separately grouped into two genetically distinct clusters. Although *M. plutonius* is known to lose virulence quickly when cultured artificially, experimental infection of representative isolates showed that atypical *M. plutonius* maintained the ability to cause EFB in honeybee larvae even after cultured *in vitro* in laboratory media. Because the rapid decrease of virulence in cultured *M. plutonius* was a major impediment to elucidation of the pathogenesis of EFB, atypical *M. plutonius* discovered in this study will be a breakthrough in EFB research.

## Introduction

European foulbrood (EFB) is an infectious and contagious bacterial disease of honeybee larvae. It affects mainly unsealed larvae and kills them at the age of 4 to 5 days. The dead larvae turn yellowish, then brown, decompose, and become watery. The larval remains often give off a foul or sour smell due to secondary invaders, such as *Enterococcus faecalis* and *Paenibacillus alvei*. EFB occurs in most areas in the world where apiculture is practiced, and is recognized as an economically important disease for apiculture.

The causative agent of EFB is a Gram-positive lanceolate coccus, *Melissococcus plutonius*. This bacterium was originally described in 1912 by White [1], and was first cultured and characterized in detail by Bailey [2]. *M. plutonius* is a fastidious organism, requiring microaerophilic to anaerobic conditions and carbon dioxide for growth. In addition, the Na∶K ratio required for growth is described to be 1 or less [3], and thus, the addition of potassium phosphate to the culture media is usually required for isolation and maintenance of this bacterium. Despite the long history of *M. plutonius* research, nothing is known about the virulence factors of this pathogen, and our understanding of the pathogenesis of EFB remains very limited. For elucidation, experimental infections of honeybee larvae with well-characterized or genetically engineered *M. plutonius* strains are needed. However, because *M. plutonius* is reported to lose its virulence quickly when subcultured in laboratory media *in vitro*, reproduction of EFB in honeybee larvae by artificially cultured *M. plutonius* is considered to be difficult. In fact, although Giersch et al. [4] transmitted EFB to honeybee larvae by feeding them with *M. plutonius* subcultured from primary cultures, it was necessary to feed a high dose of *M. plutonius* to achieve high mortality in larvae. McKee et al. [5] used a second subculture of *M. plutonius* for their experimental infections, but the disease could not be transmitted by the culture. Although Bailey [6] also tried to produce EFB in larvae with pure cultures of *M. plutonius*, the attempts failed. These difficulties largely hamper studies of the etiology of EFB.

*M. plutonius* is a one genus-one species bacterium and is thought to be remarkably homogeneous based on morphological, physiological, immunological, and genetic studies [7]–[9]. For instance, Djordjevic et al. [9] reported that Australian *M. plutonius* isolates originating from geographically diverse regions were markedly similar in their whole cell proteins, immunoreactive antigens, and DNA restriction endonuclease profiles. Moreover, they showed genetic homogeneity among Australian and British isolates and suggested that this species may be clonal [9]. However, the geographical locations of the isolates characterized so far have been mainly Europe, North and South America, and Australia. In contrast, information on *M. plutonius* isolates in other areas, including Asian countries remains limited.

In Japan, EFB is legally designated as a monitored infectious disease of honeybees and has been occurring sporadically since suspected cases were recognized in the 1980s. In Japanese cases, however, in addition to *M. plutonius* isolates with typical cultural characteristics, *M. plutonius*-like organisms have often been isolated from diseased larvae with clinical signs of EFB in regional diagnostic centers. These *M. plutonius*-like isolates are morphologically similar to *M. plutonius* and positive for *M. plutonius*-specific PCR [10] (see Materials and Methods). However, unlike typical *M. plutonius* strains, they can grow independently of the Na∶K ratio in the medium, implying that *M. plutonius* is a more heterogeneous species than reported previously. However, the taxonomic position of *M. plutonius*-like organisms was unclear. In addition, it has not been experimentally demonstrated yet whether the *M. plutonius*-like organisms can really cause EFB in honeybee larvae. In this study, we therefore investigated in detail the phenotypic characteristics of 33 *M. plutonius* and *M. plutonius*-like strain/isolates and determined their taxonomic position by molecular approaches. Moreover, we analyzed the genetic diversity of the isolates by pulsed-field gel electrophoresis (PFGE) and investigated the virulence of representative isolates by experimental infections of artificially reared honeybee larvae. Our results showed that *M. plutonius*-like organisms are taxonomically identical to *M. plutonius* and that *M. plutonius* consists of at least two groups of strains, typical and atypical *M. plutonius*, which are phenotypically and genetically distinguishable. Furthermore, atypical (*M. plutonius*-like) isolates were revealed to maintain their virulence even in *in vitro* culture and to cause EFB in honeybee larvae in experimental infections.

## Results

### Phenotypic differences between *M. plutonius* and *M. plutonius*-like organisms

As reported previously [2]–[3], [7]–[8], *M. plutonius* type strain ATCC 35311 and all typical *M. plutonius* isolates were fastidious in their culture requirements. On media supplemented with potassium phosphate (Medium 1, KBHI and KSBHI agar; Table 1), they grew well under anaerobic conditions (Table 2). However, in the absence of potassium phosphate (Medium 2, SBHI and BHI; Table 1) or when the potassium phosphate in Medium 1 was replaced with sodium phosphate (Medium 6; Table 1), growth became weak or was inhibited completely (Table 2). Although ATCC 35311 grew under anaerobic conditions on media supplemented with the other potassium salts tested (Medium 3, 4 and 5; Table 1), the other isolates did not or only grew slightly on such media. In addition, under aerobic conditions, these strain/isolates did not grow in any of the media tested (Table 2).

**Table 1:** Formulas of culture media used in this study.

	Medium 1^a^	Medium 2^b^	Medium 3^a^	Medium 4^c^	Medium 5^c^	Medium 6^d^	KSBHI agar	KBHI agar	SBHI agar	BHI agar	carbohydrate test media^a^
Agar (Difco)	15	15	15	15	15	15	15	15	15	15	2
Yeast Extract (Difco)	10	10	10	10	10	10	-	-	-	-	10
Glucose (WAKO)	10	10	10	10	10	10	-	-	-	-	-
Soluble Starch (Difco)	10	10	10	10	10	10	10	-	10	-	10
KH_2_PO_4_ e (WAKO)	13.6	-	-	-	-	-	20.4	20.4	-	-	13.6
KCl^e^ (WAKO)	-	-	7.4	-	-	-	-	-	-	-	-
K_3_(C_6_H_5_O_7_)·H_2_O^e^ (WAKO)	-	-	-	10.8	-	-	-	-	-	-	-
KHCO_3_ e (WAKO)	-	-	-	-	10	-	-	-	-	-	-
NaH_2_PO_4_·2H_2_O^e^ (WAKO)	-	-	-	-	-	15.6	-	-	-	-	-
Carbohydrate^f^ (WAKO)	-	-	-	-	-	-	-	-	-	-	10^g^
Bromocresol purple (WAKO)	-	-	-	-	-	-	-	-	-	-	0.03
Brain Heart Infusion (Difco)	-	-	-	-	-	-	37	37	37	37	-

Unit: g/L.

Medium 1 to 6 and carbohydrate test media were autoclaved at 115°C for 10 min. Other media were autoclaved at 121°C for 15 min.

a The pH was adjusted to 6.6 with KOH.

b The pH was adjusted to 6.6 with the solution, in which the mole ratio of KOH/NaOH was 1∶1.

c The pH was adjusted to 6.6 with HCl.

d The pH was adjusted to 6.6 with NaOH.

e Potassium and sodium salts were added to the media to final concentrations of 0.033 M (Medium 4), 0.1 M (Medium 1, 3, 5 and 6) or 0.15 M (KSBHI and KBHI).

f D-cellobiose, lactose, D-raffinose, or D-xylose

g After the base medium was autoclaved, the carbohydrate was added aseptically.

**Table 2:** Culture characteristics of *M. plutonius* and *M. plutonius*-like strain/isolates used in this study.

		Results for^a^		
Medium	Culture conditions	ATCC 35311	*M. plutonius* isolates	*M. plutonius*-like isolates
Medium 1	anaerobic	+^v^	+	+
	air plus 5% CO_2_	+^w^	− or +^w^	+
	aerobic	−	−	+
Medium 2	anaerobic	+^w^	− or +^w^	+ or +^v^
	air plus 5% CO_2_	−	−	+
	aerobic	−	−	+
Medium 3	anaerobic	+	+^w^	+
	air plus 5% CO_2_	+^w^	−	+
	aerobic	−	−	+
Medium 4	anaerobic	+^w^	−	+
	air plus 5% CO_2_	−	−	+
	aerobic	−	−	+
Medium 5	anaerobic	+^w^	−	+
	air plus 5% CO_2_	−	−	−
	aerobic	−	−	−
Medium 6	anaerobic	−	−	+^w^ or +
	air plus 5% CO_2_	−	−	−
	aerobic	−	−	−
KSBHI agar	anaerobic	+	+	+
	air plus 5% CO_2_	−	−, +^w^ or +	+
	aerobic	−	−	+
KBHI agar	anaerobic	+	+	+
	air plus 5% CO_2_	−	− or +^w^	+^w^ or +
	aerobic	−	−	+^w^ or +
SBHI agar	anaerobic	−	−	+
	air plus 5% CO_2_	−	−	+^w^ or +
	aerobic	−	−	−
BHI agar	anaerobic	−	−	+^v^
	air plus 5% CO_2_	−	−	+^w^ or +
	aerobic	−	−	−

a The growth of ATCC 35311 cultured on KSBHI agar under anaerobic conditions was scored as +. Compared to this growth, more vigorous and weaker growth was scored as +^v^ and +^w^, respectively. No growth or only trace levels of growth was scored as −.

In contrast, the culture requirements of *M. plutonius*-like isolates were not fastidious. Under anaerobic condition, they grew well not only on media supplemented with potassium salt (Medium 1, 3, 4, and 5, KSBHI and KBHI agars), but also on media not supplemented with potassium salt (Medium 2, SBHI and BHI) or supplemented with sodium phosphate (Medium 6). Moreover, the *M. plutonius*-like isolates grew on various media, in particular those supplemented with potassium salt, even under aerobic and air plus 5% CO_2_ conditions (Table 2).

Biochemical characteristics were also different between *M. plutonius* and *M. plutonius*-like organisms (Table 3). Although *M. plutonius* strain/isolates produced acid from glucose, fructose, and D-mannose, they did not utilize the other carbohydrates tested. In contrast, in addition to the three sugars, all *M. plutonius*-like isolates produced acids from L-arabinose, D-cellobiose, and salicin, and two (DAT557 and DAT565) also slightly produced acid from lactose. Moreover, only *M. plutonius*-like isolates, but not *M. plutonius* strain/isolates, were positive for β-glucosidase activity and hydrolyzed esculin. Furthermore, phosphatase and β-galactosidase activity of *M. plutonius*-like isolates was apparently stronger than those of *M. plutonius* strain/isolates.

**Table 3:** Biochemical characteristics of *M. plutonius* and *M. plutonius*-like strain/isolates used in this study.

	Results for		
Characteristics or tests	ATCC 35311	*M. plutonius* isolates	*M. plutonius*-like isolates
Catalase	−	−	−
Oxidase	−	−	−
Nitrate reduction	−	−	−
Production of indole	−	−	−
Urease	−	−	−
Hydrolysis of gelatin	−	−	− or ±
Phosphatase	− or ±^a^	±	+
β-Galactosidase	±	− or ±	+
β-Glucosidase	−	−	+
Hydrolysis of esculin	−	−	+
Production of acid from carbohydrate			
Glucose	+	+	+
Fructose	+	+	+
D-Mannose	+	+	+
L-Arabinose	−	−	+
D-Cellobiose	−	−	+
Salicin	−	−	+
Lactose	−	−	− or ±
Others^b^	−	−	−

a Repeated tests showed slightly different results in ATCC 35311.

b Mannitol, sucrose, maltose, D-xylose, glycerin, D-melezitose, D-raffinose, D-sorbitol, L-rhamnose, and D-trehalose.

Despite the differences in phenotypic characteristics, *M. plutonius* and *M. plutonius*-like isolates were morphologically similar. Under anaerobic conditions, both formed visible colonies on potassium phosphate-supplemented media after a few days of incubation at 37°C. The colonies were white, opaque and up to 1 mm in diameter (Figure S1). Bacterial cells in the colonies were Gram-positive lanceolate cocci, and sometimes showed rod-like forms. They occurred singly, in pairs, or in chains of varying length (Figure S2). However, compared to *M. plutonius* isolates, *M. plutonius*-like isolates formed larger colonies (Figure S1). In addition, *M. plutonius* strain/isolates sometimes formed large colony variants on KSBHI agar, but such variants were not observed in the *M. plutonius*-like isolates tested (data not shown).

### Taxonomic position of *M. plutonius*-like organisms

To determine the taxonomic position of *M. plutonius*-like organisms, we analyzed nearly complete 16S rRNA gene sequences (1,510 bp) of all strain/isolates used in this study. The sequences of both *M. plutonius* and *M. plutonius*-like isolates showed more than 99.8% homology with that of ATCC 35311, suggesting that the *M. plutonius*-like organisms also belong to *M. plutonius*. To confirm this result, we selected representative strain/isolates on the basis of the 16S rRNA gene sequences and further analyzed the levels of DNA relatedness by DNA-DNA hybridization. As shown in Table 4, reciprocal hybridization between ATCC 35311 and *M. plutonius*-like isolates, as well as between *M. plutonius* and *M. plutonius*-like isolates, showed more than 80% DNA relatedness, whereas DNA relatedness between *M. plutonius*-like isolates and *E. faecalis* type strain NCTC 775 was only approximately 10%. Because a value of 70% was proposed as the recommended standard for delineating species [11], these results confirmed that *M. plutonius*-like organisms were taxonomically identical to *M. plutonius*. We hereafter refer to *M. plutonius* and *M. plutonius*-like organisms as typical and atypical *M. plutonius*, respectively.

**Table 4:** Results of DNA-DNA hybridization.

	% Similarity with labeled DNA from:^a^							
Immobilized DNA	ATCC 35311	DAT351	DAT561	DAT565	DAT585	DAT606	DAT607	NCTC 775
ATCC 35311	100.0	99.5	94.2	85.5	95.7	97.7	94.1	8.9
DAT351^b^	96.4	100.0	97.8	86.4	89.6	97.1	94.4	9.2
DAT561^b^	94.4	98.9	100.0	88.7	82.1	95.2	95.9	8.5
DAT565^b^	101.5	109.2	101.0	100.0	94.3	99.9	99.9	10.6
DAT585^c^	101.7	98.8	95.8	85.9	100.0	100.0	91.4	9.0
DAT606^c^	107.0	107.2	100.3	87.9	97.3	100.0	93.8	9.7
DAT607^b^	104.9	112.1	106.6	96.3	96.4	98.8	100.0	9.0
NCTC 775^d^	11.6	12.9	10.9	10.1	11.2	13.4	11.9	100.0

a Because % similarities were shown as the degree of DNA-DNA reassociation calculated based on OD_405_ values obtained as the result of enzyme reaction, they can be greater than 100%.

b *M. plutonius*-like isolates.

c *M. plutonius* isolates.

d *E. faecalis* type strain.

It is noteworthy that, except for DAT585, which had a single nucleotide difference, the 16S rRNA gene sequences of all typical *M. plutonius* strain/isolates including ATCC 35311 were identical regardless of the geographical source and year of isolation. On the other hand, the sequences of all atypical *M. plutonius* isolates had two or three nucleotide differences compared to the type strain. In addition, even among the atypical *M. plutonius* isolates isolated in the same area in recent years, several nucleotide differences were observed (data not shown).

### Genetic diversity of *M. plutonius* isolates

**Figure 1:**
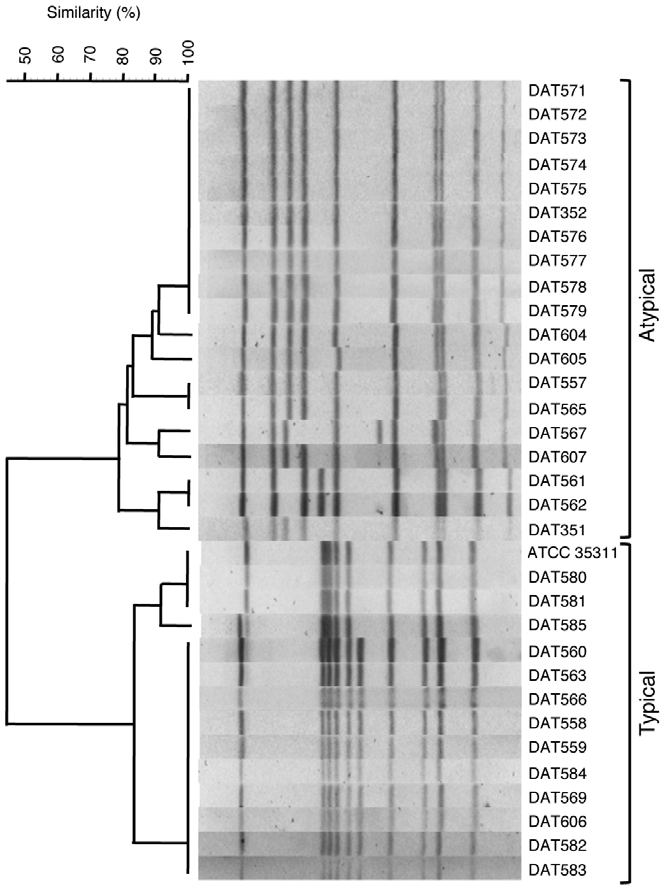
Dendrogram of SmaI-digested PFGE profiles of typical and atypical *M. plutonius* strain/isolates. Phenotypically distinct strain/isolates were also grouped separately into two distinct genetic clusters.

PFGE analysis of SmaI-digested genomic DNA showed that ATCC 35311 and typical *M. plutonius* isolates had similar PFGE profiles (more than 83.77% similarity) and formed a single PFGE cluster (Figure 1). Among them, ATCC 35311 isolated in UK and two Japanese isolates shared an identical PFGE profile, and DAT569 isolated in Paraguay and nine Japanese isolates had another identical PFGE profile. Atypical *M. plutonius* isolates also had similar PFGE profiles (more than 78.81% similarity) and formed another single cluster (Figure 1). However, the profiles were clearly different from those of typical *M. plutonius*, demonstrating that phenotypically distinct *M. plutonius* isolates have distinct genetic backgrounds.

### Experimental reproduction of EFB by atypical *M. plutonius* cultured *in vitro*

Although *M. plutonius* is well proven to be the causative agent of EFB [12], it has not been demonstrated experimentally that atypical *M. plutonius* found in this study can also cause this disease. However, because *M. plutonius* is considered to lose its virulence quickly when cultured artificially in laboratory media *in vitro* [5]–[6], experimental reproduction of EFB by well-characterized isolates is extremely difficult. To investigate whether *M. plutonius* isolates characterized in this study still show virulence in honeybee larvae, we performed experimental infections of artificially reared honeybee larvae using representative typical and atypical *M. plutonius* isolates subcultured approximately six times.

**Figure 2:**
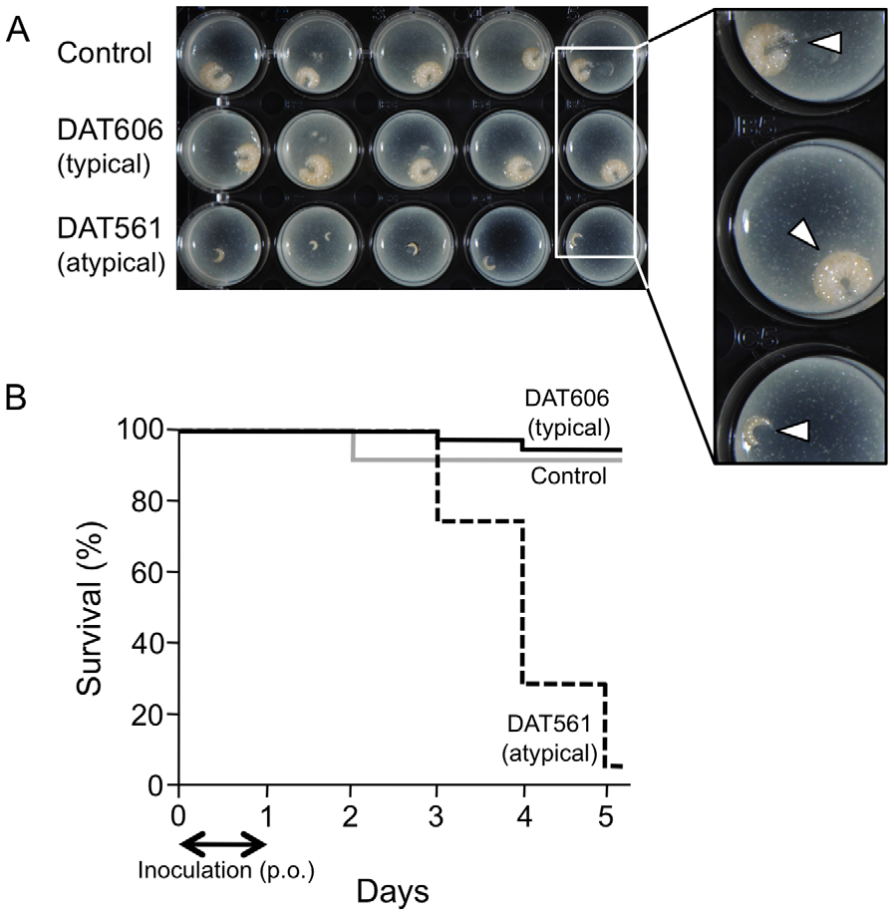
Experimental Infection of Honeybee Larvae. A) Larvae at day 4 (5 days of age). Larvae were transferred onto the surface of an excess amount of artificial diet in 24-well cell culture plates for taking photographs. Arrowheads indicate larvae. B) Survival of larvae in control (gray solid line), DAT561-inoculated (dashed line) and DAT606-inoculated (black solid line) groups. Larvae in DAT561- and DAT606-inoculated groups were fed with 10 µl artificial diet containing *M. plutonius* at a final concentration of approximately 5×10^6^ CFU/ml (approximately 5×10^4^ CFU/larva) for the first 24 h. Thirty-five larvae were used to calculate survival rates in each group, and survival rates of control, DAT561-inoculated and DAT606-inoculated groups at day 5 were 91.4%, 5.7% and 94.3%, respectively.

Artificially cultured typical *M. plutonius* isolate DAT606 did not cause EFB at all. Although several larvae died due to mechanical damage, all survived larvae were well grown (Figure 2A), and the mortality was comparable to that in the control group (log-rank test, *P* = 1) (Figure 2B). On the other hand, when larvae were fed with a diet containing artificially cultured atypical *M. plutonius* isolate DAT561, all larvae stopped growing at day 2 or 3 (Figure 2A), their respiration became slow, and 94.3% of larvae died within 5 days (i.e. <6 days of age) (Figure 2B). Mortality was significantly higher than in control and DAT606-inoculated groups (log-rank test, *P*<0.0001). Similar to EFB cases in the field, dead larvae lost body elasticity and became yellowish and watery. Similar results were also observed when other typical (DAT583 and DAT585) and atypical (DAT351 and DAT573) isolates were used (Figure S3).

To confirm *M. plutonius* infection in larvae, we randomly selected more than 11 larvae from each group and isolated *M. plutonius* on KSBHI agar. As expected, no bacteria were isolated from larvae in the control group. In contrast, a large number of atypical *M. plutonius* were isolated in pure culture from dead larvae in the DAT561-inoculated group (1.00×10^6^ to 2.93×10^7^ CFU/larva). Interestingly, numerous typical *M. plutonius* were also isolated in pure culture from all well-grown larvae in the DAT606-inoculated group tested (5.88×10^6^ to 1.65×10^8^ CFU/larva). Except for two cases in which a single *M. plutonius* colony was isolated from 10 µl leftover artificial diet for DAT606-inoculated larvae, no bacteria were isolated from the diet tested, indicating that isolation of *M. plutonius* did not result from cross-contamination from the diet but from the infection of larvae with *M. plutonius*. These results demonstrated that atypical *M. plutonius* has the ability to cause EFB in honeybee larvae and that, unlike typical *M. plutonius*, virulence can be maintained even after repeated subculture in laboratory media.

## Discussion

Honeybees are not only important for the honey and bee products they produce but are also vital pollinators of agricultural crops; hence, infectious diseases of honeybees including EFB have great economic impact worldwide. Despite their importance, our understanding of *M. plutonius* remains very limited. Although *M. plutonius* has been thought to be homologous or clonal [7]–[9], our results demonstrated that this species consists of at least two groups of strains (typical and atypical *M. plutonius*) that are phenotypically and genetically distinguishable. Interestingly, irrespective of the geographical source and year of isolation, all typical *M. plutonius* strain/isolates showed very similar PFGE profiles. In addition, except for one isolate, they had identical 16S rRNA gene sequences. On the other hand, even among the recent Japanese isolates, PFGE profiles of typical and atypical *M. plutonius* were obviously different. These results suggest that the significantly different characteristics observed in typical and atypical *M. plutonius* were not adaptation to laboratory media but were traits present in natural populations of *M. plutonius*.

The genetic background responsible for cultural and biochemical differences between typical and atypical isolates is unknown. Recently, genome sequencing of *M. plutonius* type strain ATCC 35311 was completed [13]. The genome consisted of a single circular chromosome of approximately 1.9 Mbp and a large plasmid of approximately 178 kbp. Intriguingly, ATCC 35311 harbored only two genes putatively involved in potassium metabolism [13] while, according to the automated annotation server RAST [14], *E. faecalis* V583, the closest organism to *M. plutonius* ATCC 35311 in the database, has at least 10 genes putatively participating in the function (unpublished observation), suggesting that a lack of genes associated with potassium metabolism might be involved in the potassium requirement for the growth of typical *M. plutonius*. Although genome sequence data of atypical *M. plutonius* strains are not yet available, further comparative genomic analysis will provide novel insights into the physiology of *M. plutonius*.

McKee et al. [5] successfully transmitted EFB to artificially reared honeybee larvae by feeding them with *M. plutonius* directly extracted from diseased larvae. However, when they used artificially cultured *M. plutonius* (second subculture), EFB did not develop even in larvae fed 1.1×10^9^ organisms/ml continuously [5]. Difficulties in inducing EFB by cultured *M. plutonius* were also reported by Bailey [6], and these difficulties were considered to be due to the rapid decrease of *M. plutonius* virulence in culture media. In accordance with these reports, cultured typical *M. plutonius* isolates tested in this study did not cause EFB in honeybee larvae. In contrast and surprisingly, although the number of subcultures performed before experimental infections was almost the same in both typical and atypical isolates (approximately six times), cultured atypical *M. plutonius* isolates killed most of the infected larvae within 5 days (Figures 2 and S3). Dead larvae showed typical clinical signs of EFB and a number of atypical *M. plutonius* were isolated from all the larvae tested, demonstrating that atypical *M. plutonius* is certainly a causative agent of EFB and can maintain its virulence in laboratory media. Because all *M. plutonius* isolates used in this study were isolated from diseased larvae with typical clinical signs of EFB and clinical signs of diseased larvae, from which typical and atypical *M. plutonius* were isolated, were indistinguishable in field cases (unpublished observations), typical *M. plutonius* is also expected to have the ability to cause EFB *in vivo*. Because Bailey showed increased virulence of *M. plutonius* following passage of the organism through a larval cycle [15], the expression of genes involved in the virulence might be negatively regulated under artificial culture conditions in typical *M. plutonius*. Alternatively, because it was shown that both *M. plutonius* and *P. alvei* (a common secondary invader associated with EFB) were important in the development of EFB in honeybee larvae [4], the presence of secondary invaders, such as *P. alvei* or substances from the secondary invaders might be necessary for typical *M. plutonius* to express virulence genes. In contrast, atypical *M. plutonius* might be able to express virulence genes even under artificial conditions and/or the absence of any secondary invaders. However, it is still unknown how atypical isolates killed larvae. In nature, *M. plutonius* multiplies only within the larval gut of the honeybee and Bailey suggested that the pathogenic effect associated with EFB was the competition of food resources between larva and bacteria and the consequent starvation of larvae [12]. However, McKee et al. provided larvae with excess food in their experiments and yet disease symptoms and death of larvae occurred in the groups fed with *M. plutonius* extracted from diseased larvae [5]. In our experiments, we also gave sufficient food to atypical *M. plutonius*-inoculated larvae; nevertheless, their growth stopped within a few days (Figure 2A). By contrast, despite the heavy infection of *M. plutonius*, typical isolate-inoculated larvae grew very well (Figure 2A). These results indicate the presence of additional pathogenic mechanisms, as speculated previously [5]. Comparison of gene expressions in artificially cultured typical and atypical isolates will give important clues for elucidating the pathogenic mechanisms of EFB.

It is noteworthy that Giersch et al. [4] transmitted EFB to artificially raised honeybee larvae using *M. plutonius* subcultured from primary cultures derived from diseased larval smears, although 1×10^7^ infective units/larva (each chain of *M. plutonius* cells observed microscopically was counted as a single infective unit) was required to obtain more than 80% mortality at 169 h post grafting. It is unknown why artificially cultured *M. plutonius* killed larvae in the experiments, but not in other previous studies [5]–[6]. Because *M. plutonius* seems to have been subcultured only once in the study [4], inoculated isolates might still express virulence genes. Alternatively, although *M. plutonius* isolates used by Giersch et al. [4] were not characterized, they might also be isolates that can maintain virulence *in vitro*.

Previous studies have also reported several unusual or non-fastidious putative *M. plutonius* strain/isolates: an English strain, which grew on medium not supplemented with potassium phosphate [8]; Brazilian isolates, which grew moderately even on medium supplemented with sodium phosphate [7], [16]; and an Indian isolate from *Apis cerana*, the growth of which was extremely inhibited under high CO_2_ concentrations on medium supplemented with standard Difco yeast extract [17]. Although the basal media used in previous studies were not the same as those used in this study, culture characteristics of the Brazilian isolates were relatively similar to those of the Japanese atypical isolates. However, in Brazilian isolates, no or only feeble growth was observed when the isolates were incubated aerobically [7], [16], whereas the Japanese atypical isolates grew under aerobic conditions on many media tested, in particular those containing potassium salts (Table 2). In addition, some biochemical characteristics including maltose utilization seemed different between the Brazilian and Japanese isolates [7], suggesting that they are not identical. Because the previously reported strain/isolates have not been analyzed by molecular approaches, including DNA-DNA hybridization and PFGE, their taxonomic positions and relationships to Japanese atypical isolates are unclear. However, these reports and our present observation imply further phenotypic and genetic heterogeneity in the population of *M. plutonius*.

In Japan, native honeybees (Japanese honeybee, *Apis cerana japonica*) live in various parts of the country and have been kept by traditional methods for hundreds of years, whereas the European honeybee (*A. mellifera*), which is now commonly used in apiculture throughout the world, was imported into Japan in 1877 in order to increase honey productivity. Therefore, marked homology among typical *M. plutonius* isolated in different countries and significant differences between typical and atypical isolates observed in this study may suggest that typical and atypical *M. plutonius* became separate types long ago perhaps when the two species of honeybee became distinct, and that typical *M. plutonius* was introduced into Japan recently with *A. mellifera*, whereas atypical *M. plutonius* was prevalent in Japan with *A. cerana japonica* and transmitted to imported *A. mellifera* recently. However, although *M. plutonius* has been reported to be isolated not only from *A. mellifera*, but also from *A. cerana* [17] and *Apis laboriosa* [18], we have not yet isolated *M. plutonius* from *A. cerana japonica*. It is also unknown whether the atypical *M. plutonius* reported in this study is unique to Japan or widely distributed in neighboring countries. To investigate the above hypothesis, therefore, further studies examining native honeybees and a range of geographically diverse, international isolates will be required.

In conclusion, we demonstrated that *M. plutonius* is a more heterogeneous species than believed so far and that artificially cultured atypical *M. plutonius* isolates can cause EFB in honeybee larvae. To our knowledge, the atypical isolates found in this study are the first *M. plutonius* that can maintain virulence even after repeated subculture *in vitro*, so our discovery is a major breakthrough for future research on the pathogenesis of this important honeybee disease.

## Materials and Methods

### Bacterial strain/isolates

Thirty-three *M. plutonius* and *M. plutonius*-like strain/isolates were used in this study (Table 5). Thirty-one isolates (12 *M. plutonius* and 19 *M. plutonius*-like) were isolated in various areas of Japan and two were isolated in the UK (type strain ATCC 35311) and Paraguay (DAT569). ATCC 35311 was purchased from the American Type Culture Collection, and the others were isolated from diseased larvae of *A. mellifera* with clinical signs of EFB. All the strain/isolates were positive for *M. plutonius*-specific PCR [10] (Figure S4).

**Table 5:** Strain/isolates used in this study.

Strain/Isolates^a^	Country/Area	Year	Accession no. of 16S rRNA gene sequence
*M. plutonius* (typical *M. plutonius*)			
ATCC 35311^b^	UK	before 1982	AB614100
DAT558	Kanto region, Japan	2008	AB614071
DAT559	Kanto region, Japan	2009	AB614072
DAT560	Kanto region, Japan	2009	AB614073
DAT563	Kanto region, Japan	2009	AB614076
DAT566	Kanto region, Japan	2008	AB614078
DAT569	Paraguay	1993 or 1994	AB614094
DAT580	Chugoku region, Japan	1990	AB614089
DAT581	Chugoku region, Japan	1991	AB614090
DAT582	Chugoku region, Japan	1992	AB614091
DAT583	Chugoku region, Japan	1992	AB614092
DAT584	Chugoku region, Japan	1995	AB614093
DAT585	Kanto region, Japan	1988	AB614095
DAT606	Kanto region, Japan	2010	AB614098
*M. plutonius*-like (atypical *M. plutonius*)			
DAT351	Chubu region, Japan	2004	AB614069
DAT352	Chubu region, Japan	2006	AB614068
DAT557	Kanto region, Japan	2005	AB614070
DAT561	Kanto region, Japan	2009	AB614074
DAT562	Kanto region, Japan	2009	AB614075
DAT565	Kanto region, Japan	2005	AB614077
DAT567	Tohoku region, Japan	2009	AB614079
DAT571	Chugoku region, Japan	1991	AB614080
DAT572	Chugoku region, Japan	1991	AB614081
DAT573	Chugoku region, Japan	1991	AB614082
DAT574	Chugoku region, Japan	1991	AB614083
DAT575	Chugoku region, Japan	1991	AB614084
DAT576	Chugoku region, Japan	1991	AB614085
DAT577	Chugoku region, Japan	1991	AB614086
DAT578	Chugoku region, Japan	1991	AB614087
DAT579	Chubu region, Japan	before 1997	AB614088
DAT604	Kanto region, Japan	2003	AB614096
DAT605	Kanto region, Japan	2006	AB614097
DAT607	Kanto region, Japan	2010	AB614099

a All strain/isolates were isolated from diseased larvae of European honeybee (*A. mellifera*) and were positive for *M. plutonius*-specific PCR (Figure S4).

b Type strain.

### Culture media and growth conditions

The formulas of the culture media used in this study are shown in Table 1. Medium 1 was recommended for isolation of *M. plutonius* in Bergey's Manual of Systematic Bacteriology [19]. Medium 2 to 6 were modified on the basis of Medium 1. KSBHI, KBHI and SBHI agar were brain heart infusion (BHI; Difco Laboratories, Becton Dickinson, Sparks, MD)-based media supplemented with 0.15 M KH_2_PO_4_ plus 1% soluble starch, 0.15 M KH_2_PO_4_ and 1% soluble starch, respectively. For cultural characterization, bacteria were cultured on various media at 37°C for a week under aerobic, air plus 5% CO_2_ and anaerobic conditions using the Anaero Pack System (Mitsubishi Gas Chemical Co., Inc., Tokyo, Japan). The growth of ATCC 35311 cultured on KSBHI agar under anaerobic condition was scored as +. Compared to this growth, more vigorous and weaker growth was scored as +^v^ and +^w^, respectively. No growth or only trace levels of growth were scored as −. Unless otherwise stated, bacteria were cultured on Medium 1 or KSBHI agar at 37°C under anaerobic conditions for other analyses.

### Biochemical tests

Conventional methods were used to test for the production of catalase and oxidase. Other biochemical tests were performed using API 20A (bioMerieux, Marcy l'Etoile, France), Rapid ID 32A (bioMerieux) and ID-Test HN-20 (Nissui Pharmaceutical Co., Ltd., Tokyo, Japan). The results of acid production from D-cellobiose, lactose, D-raffinose and D-xylose were further confirmed by conventional methods using carbohydrate test media (Table 1) after two weeks incubation of bacteria at 37°C under anaerobic conditions.

### Genomic DNA extraction

Bacterial cells were harvested from KSBHI agar, suspended in 500 µl TE 50∶5 [50 mM Tris-HCl (pH 8.0), 5 mM EDTA (pH 8.0)] containing 5 mg/ml lysozyme and 40 U/ml mutanolysin, and incubated for 1 h at 37°C. After the addition of 20 µl of 10% sodium dodecyl sulfate, the mixtures were extracted with an equal volume of phenol, phenol-chloroform-isoamyl alcohol (25∶24∶1) (PCI) and chloroform at least once, three times and once, respectively. Nucleic acid was then precipitated by ethanol, rinsed with 70% ethanol and suspended in 100 µl TE [10 mM Tris-HCl (pH 8.0), 1 mM EDTA (pH 8.0)]. For DNA-DNA hybridization, the extracted DNA was further treated with 50 µg/ml RNase at 37°C for 1 hour and then 200 µg/ml proteinase K at 37°C for 1 hour, extracted with PCI and chloroform, and precipitated by ethanol. When necessary, the protocol was scaled up according to sample size.

### 16S rRNA gene sequence analysis

The 16S rRNA genes of *M. plutonius* and *M. plutonius*-like organisms were amplified from the genomic DNA by *Ex* Taq polymerase (Takara Bio, Otsu, Japan) using primers F1 (5′-GAGTTTGATCCTGGCTCAG-3′) and R13 (5′-AGAAAGGAGGTGATCCAGCC-3′) [20]. The amplified fragments were sequenced by a BigDye terminator v3.1 cycle sequencing kit using a 3130xl Genetic Analyzer (Applied Biosystems, Tokyo, Japan). Sequencher Ver. 4.8 (Hitachi Software Engineering Co., Ltd., Yokohama, Japan), the CLUSTALW Ver. 1.83 (http://clustalw.ddbj.nig.ac.jp/top-e.html) and the BLAST programs (http://www.ncbi.nlm.nih.gov/BLAST) were used for assembly and analysis of the sequences.

### DNA-DNA hybridization

DNA-DNA hybridization was carried out under stringent conditions (37°C) according to the microplate method [21]–[22] with appropriate modifications. Bacterial DNA or calf thymus DNA (negative control) was immobilized in microdilution wells by incubating the wells with 100 µl denatured DNA [20 µg/ml in phosphate-buffered saline (PBS) containing 0.1 M MgCl_2_] at 30°C for 16 h. After removal of the solution, the wells were dried and incubated with 200 µl prehybridization solution (2× SSC, 2× Denhardt's solution, 50% formamide, 40 µg/ml denatured calf thymus DNA) at 37°C for 30 min. The solution was then replaced with 100 µl hybridization solution [prehybridization solution containing 3 µg/ml denatured bacterial DNA labeled with photobiotin (Vector Laboratories, Inc., Burlingame, CA.) according to the manufacturer's instructions]. After hybridization at 37°C for 16 h, the wells were washed three times with 1× SSC and incubated with 100 µl streptavidin-alkaline phosphatase solution [Histofine (Nichirei, Tokyo, Japan) diluted 10 times with PBS containing 1% bovine serum albumin] at 37°C for 30 min. After washing three times with 1× SSC, the wells were filled with 100 µl *p*-nitrophenylphosphate solution (KPL, Gaithersburg, MD) and incubated at room temperature. The optical density of each well at 405 nm (OD_405_) was measured within 30 min. The value of negative control wells was subtracted from that of the other wells, and the value of wells, in which immobilized DNA was hybridized with DNA of the same strain, was calculated as 100%.

### PFGE

For genomic DNA extraction, bacterial cells on KSBHI agar were harvested, washed with Tris-saline buffer [10 mM Tris-HCl (pH 8.0), 1 M NaCl], and suspended in EDTA–sarcosine buffer (6 mM Tris-HCl, 1 mM NaCl, 100 mM EDTA, 1% sodium N-lauroylsarcosine; pH 7.6). The suspension was mixed with an equal volume of 1.0% SeaKem Gold agarose (Lonza, Rockland, ME) in EDTA–sarcosine buffer and allowed to solidify in a 0.7-mm sample plug caster (Bio-Rad Laboratories, Hercules, CA). The sample plugs were incubated in lysis buffer [0.5 M EDTA (pH 8.0), 2.5 mg/ml lysozyme, 10 U/ml mutanolysin] for 3 h at 37°C, followed by incubation in proteinase K solution [0.5 M EDTA (pH 8.0), 1% sodium N-lauroylsarcosine, 1 mg/ml proteinase K] for 18 h at 50°C. The samples were then treated twice with 1 mM Pefabloc SC (Roche Applied Science, Basel, Switzerland) in TE for 30 min at 50°C and washed three times with TE at 4°C.

Genomic DNA contained in each plug was then incubated in restriction enzyme buffer for 30 min at 4°C, digested with 20 U SmaI (Takara Bio) for 18 h at 30°C and separated on a 1.0% SeaKem Gold agarose gel in 0.5× TBE buffer (44.5 mM Tris, 44.5 mM boric acid, 1 mM EDTA; pH 8.0) supplemented with 50 µM thiourea using a CHEF-DR II System (Bio-Rad Laboratories). The condition for electrophoresis was 6 V/cm with pulse times of 2.9–17.3 s for 20 h at 15°C. The bands were visualized by staining with ethidium bromide, and digital images of gels were imported into BioNumerics software, version 5.1 (Applied Maths, Sint-Martens-Latem, Belgium). A dendrogram was produced using the unweighted pair group method with the average linkage algorithm. The value of Dice predicted the similarity of two patterns at settings of 1% optimization and 1% position tolerance.

### Experimental infection of honeybee larvae

Honeybee larvae were reared *in vitro* according to the method [23] with minor modifications. Larvae were collected from *A. mellifera* colonies in a disease-free apiary at the Honeybee Research Unit, National Institute of Livestock and Grassland Science, Tsukuba. The queen was confined in an excluder cage for one day to lay eggs, and larvae (<24 h of age) were collected from the cage on the fourth day. The larvae were then grafted onto the surface of approximately 300 µl artificial diet (50% royal jelly, 37% sterile distilled water, 6% D-glucose, 6% D-fructose and 1% yeast extract) in a 35 mm diameter cell culture plate. After counting the larvae, they were divided into control and experimental groups and immediately used for experimental infection. *M. plutonius* cultured anaerobically on KSBHI agar plates at 35°C for approximately one week were collected, washed twice with saline and suspended in saline. Experimental groups of larvae were fed in 24-well cell culture plates (one larva/well) for 24 h with 10 µl artificial diet diluted with *M. plutonius* suspension in saline (1∶1) at a final concentration of approximately 5×10^6^ CFU/ml (approximately 5×10^4^ CFU/larva), whereas the control groups were fed with 10 µl artificial diet diluted with the same volume of saline. The culture plates were kept in a plastic box and incubated at 35°C with relative humidity of 90%. The larvae were moved to fresh artificial diet (10 µl/larva at days 1 to 3 and then increased according to larval growth) every day and the mortality of larvae was observed under a stereomicroscope. Dead larvae were distinguished by the lack of respiration, loss of body elasticity and color change to yellowish. More than 24 larvae were used to calculate the survival rate in each group and the experiment was performed for 5 days. For confirmation of *M. plutonius* infection, larvae were randomly selected from each group, and *M. plutonius* were isolated from the larvae on KSBHI agar plates. Differences in the survival rate of larvae were statistically analyzed by the log-rank test. For comparison, *P*<0.05 was considered significant.

### Nucleotide sequence accession numbers

The 16S rRNA gene sequences determined in this study have been deposited in the DDBJ/EMBL/GenBank database under the accession numbers listed in Table 5.

## Supporting Information

Figure S1 **Colony morphology of** ***M. plutonius*** **(DAT606) and** ***M. plutonius*** **-like (DAT571) isolates.** Bacteria were cultured under anaerobic conditions at 37°C.
(PDF)

Figure S2 **Gram staining of** ***M. plutonius*** **(DAT606) and** ***M. plutonius*** **-like (DAT571) isolates.** Bacteria were cultured under anaerobic conditions at 37°C for 5 days. Images were taken at ×1,000 magnification.
(PDF)

Figure S3 **Experimental Infection of Honeybee Larvae.** Survival of larvae in control and *M. plutonius* (DAT351, DAT573, DAT583 and DAT585)-inoculated groups. Larvae in inoculated groups were fed with 10 µl artificial diet containing *M. plutonius* at a final concentration of approximately 5×10^6^ CFU/ml (approximately 5×10^4^ CFU/larva) for the first 24 h. Thirty-five (control) or twenty-four (inoculated groups) larvae were used to calculate survival rates, and survival rates of control, DAT351-inoculated, DAT573-inoculated, DAT583-inoculated and DAT585-inoculated groups at day 5 were 91.4%, 8.3%, 29.2%, 87.5% and 83.3%, respectively. No significant differences (log-rank test, *P* = 1) in survival were observed between control and typical *M. plutonius* (DAT583 and DAT585)-inoculated groups, whereas significant differences (log-rank test, *P*<0.05) in survival were observed between atypical *M. plutonius* (DAT351 and DAT573)-inoculated and control/typical *M. plutonius* (DAT583 and DAT585)-inoculated groups.
(PDF)

Figure S4 ***M. plutonius*** **-specific PCR.** *M. plutonius*-specific PCR was performed as described previously [10] using genomic DNA extracted from *M. plutonius* and *M. plutonius*-like isolates. Genomic DNA of *M. plutonius* type strain ATCC 35311 was used as a positive control. A PCR product (0.83 kb) was amplified from all strain/isolates used in this study.
(PDF)
