# Supplementary material for: Diversity of Melissococcus plutonius from Honeybee Larvae in Japan and Experimental Reproduction of European Foulbrood with Cultured Atypical Isolates
Source: PLoS One. 2012 Mar 19;7(3):e33708. doi: 10.1371/journal.pone.0033708 (PMC3307753; doi:10.1371/journal.pone.0033708)

Figure S1. Colony morphology of *M. plutonius* (DAT606) and *M. plutonius*-like (DAT571) isolates. Bacteria were cultured under anaerobic conditions at 37°C .

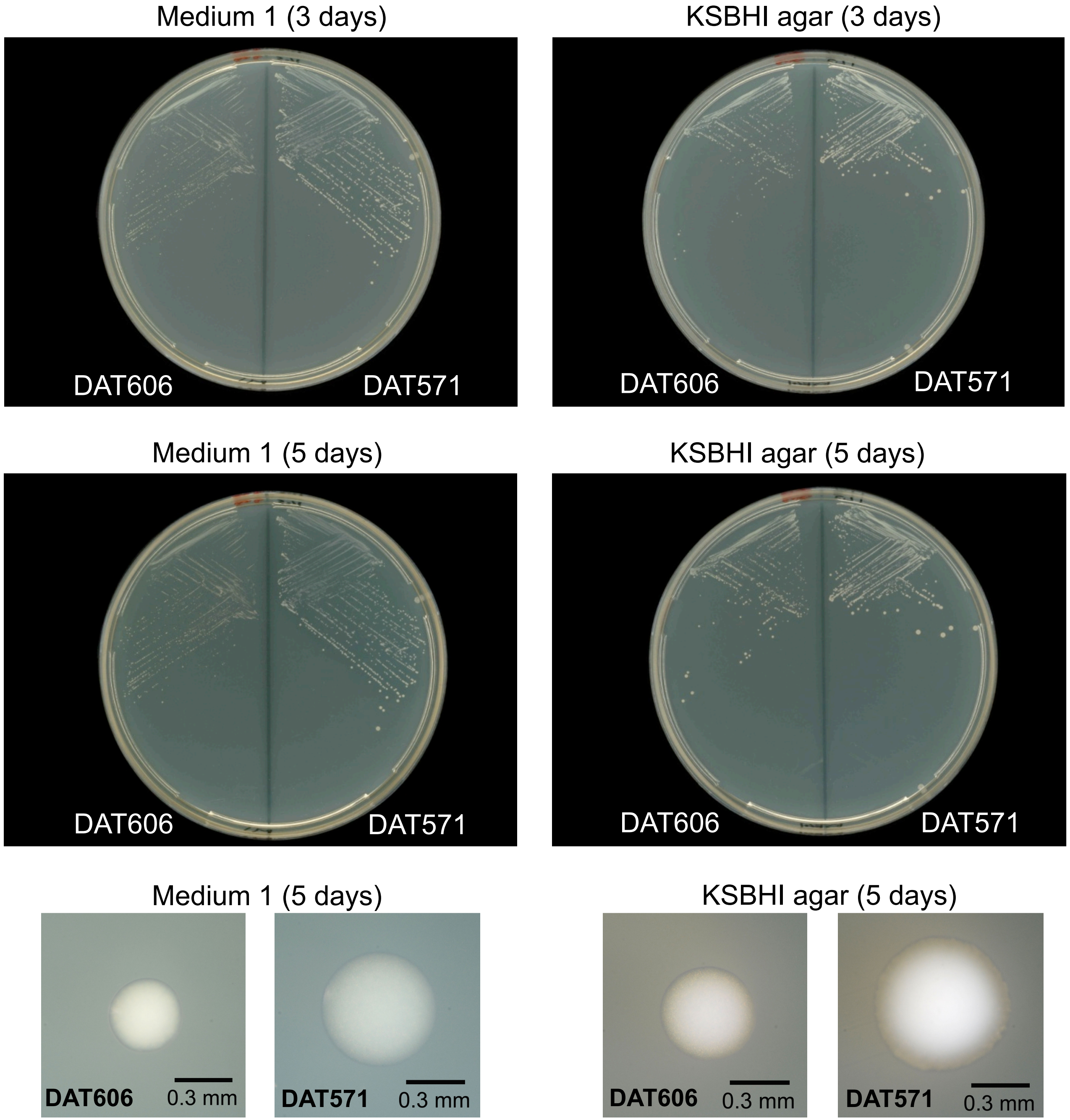

Supplement: Figure S1 — Colony morphology of M. plutonius (DAT606) and M. plutonius -like (DAT571) isolates. Bacteria were cultured under anaerobic conditions at 37°C. (PDF) [file pone.0033708.s001.pdf]
