# Supplementary material for: Diversity of Melissococcus plutonius from Honeybee Larvae in Japan and Experimental Reproduction of European Foulbrood with Cultured Atypical Isolates
Source: PLoS One. 2012 Mar 19;7(3):e33708. doi: 10.1371/journal.pone.0033708 (PMC3307753; doi:10.1371/journal.pone.0033708)
Supplement: Figure S3 — Experimental Infection of Honeybee Larvae. Survival of larvae in control and M. plutonius (DAT351, DAT573, DAT583 and DAT585)-inoculated groups. Larvae in inoculated groups were fed with 10 µl artificial diet containing M. plutonius at a final concentration of approximately 5×10^6^ CFU/ml (approximately 5×10^4^ CFU/larva) for the first 24 h. Thirty-five (control) or twenty-four (inoculated groups) larvae were used to calculate survival rates, and survival rates of control, DAT351-inoculated, DAT573-inoculated, DAT583-inoculated and DAT585-inoculated groups at day 5 were 91.4%, 8.3%, 29.2%, 87.5% and 83.3%, respectively. No significant differences (log-rank test, P = 1) in survival were observed between control and typical M. plutonius (DAT583 and DAT585)-inoculated groups, whereas significant differences (log-rank test, P<0.05) in survival were observed between atypical M. plutonius (DAT351 and DAT573)-inoculated and control/typical M. plutonius (DAT583 and DAT585)-inoculated groups. (PDF) [file pone.0033708.s003.pdf]

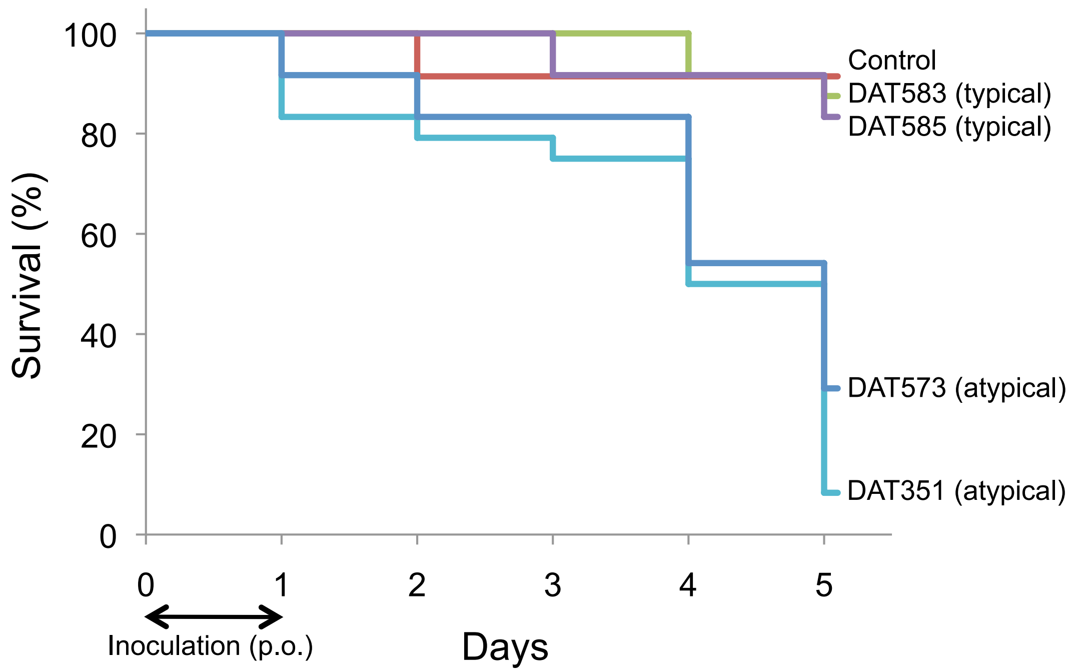

Figure S3. Experimental Infection of Honeybee Larvae. Survival of larvae in control and *M. plutonius* (DAT351, DAT573, DAT583 and DAT585)-inoculated groups. Larvae in inoculated groups were fed with 10 μl artificial diet containing *M. plutonius* at a final concentration of approximately 5×10^6 CFU/ml (approximately 5×10^4 CFU/larva) for the first 24 h. Thirty-five (control) or twenty-four (inoculated groups) larvae were used to calculate survival rates, and survival rates of control, DAT351-inoculated, DAT573-inoculated, DAT583-inoculated and DAT585-inoculated groups at day 5 were 91.4%, 8.3%, 29.2%, 87.5% and 83.3%, respectively. No significant differences (log-rank test, $P = 1$) in survival were observed between control and typical *M. plutonius* (DAT583 and DAT585)-inoculated groups, whereas significant differences (log-rank test, $P < 0.05$) in survival were observed between atypical *M. plutonius* (DAT351 and DAT573)-inoculated and control/typical *M. plutonius* (DAT583 and DAT585)-inoculated groups.
