# Supplementary material for: Diversity of Melissococcus plutonius from Honeybee Larvae in Japan and Experimental Reproduction of European Foulbrood with Cultured Atypical Isolates
Source: PLoS One. 2012 Mar 19;7(3):e33708. doi: 10.1371/journal.pone.0033708 (PMC3307753; doi:10.1371/journal.pone.0033708)
Supplement: Figure S4 — M. plutonius -specific PCR. M. plutonius-specific PCR was performed as described previously [10] using genomic DNA extracted from M. plutonius and M. plutonius-like isolates. Genomic DNA of M. plutonius type strain ATCC 35311 was used as a positive control. A PCR product (0.83 kb) was amplified from all strain/isolates used in this study. (PDF) [file pone.0033708.s004.pdf]

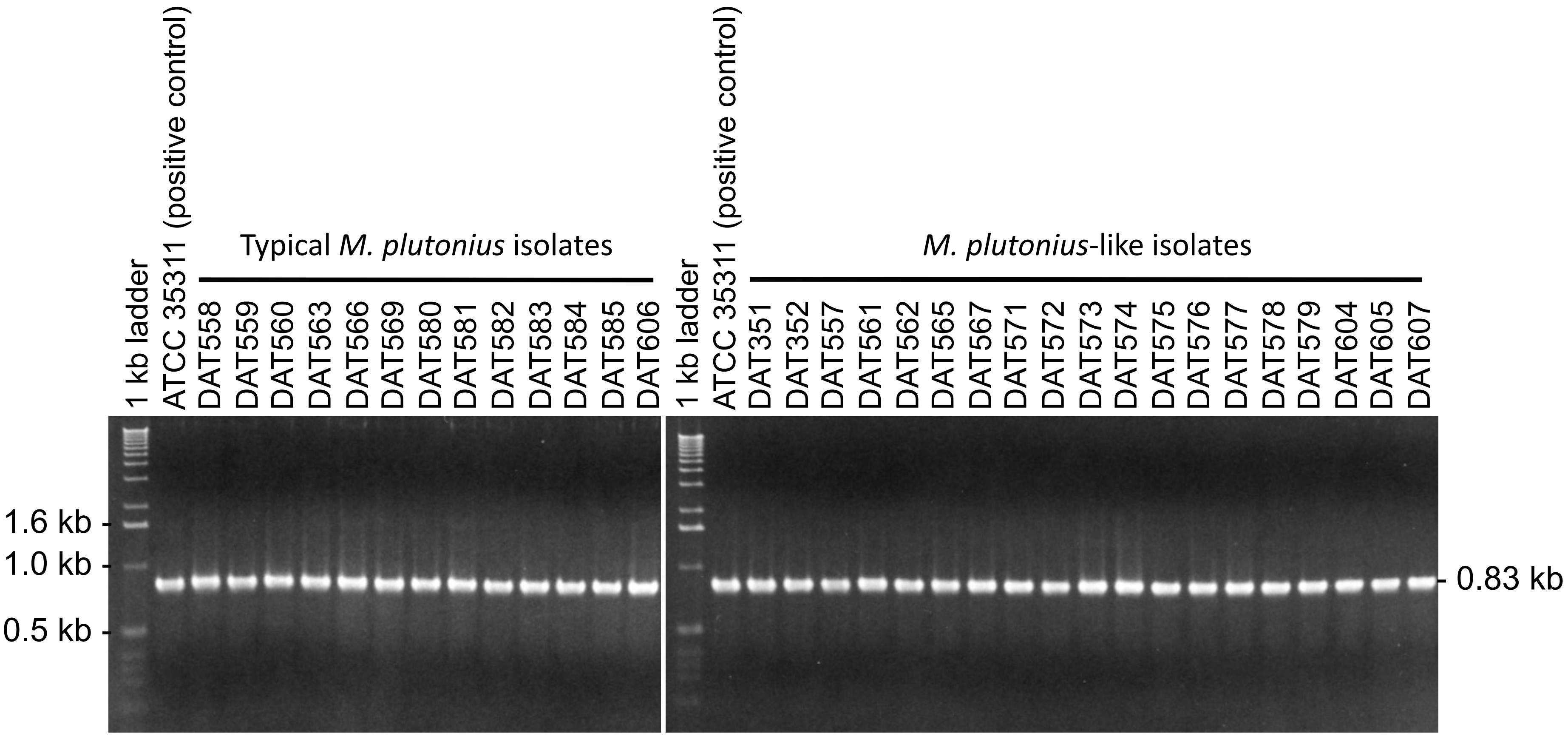

Figure S4. *M. plutonius*-specific PCR. *M. plutonius*-specific PCR was performed as described previously [10] using genomic DNA extracted from *M. plutonius* and *M. plutonius*-like isolates. Genomic DNA of *M. plutonius* type strain ATCC 35311 was used as a positive control. A PCR product (0.83 kb) was amplified from all strain/isolates used in this study.
